# The safety and utility of the semi-sitting position for clipping of posterior circulation aneurysms

**DOI:** 10.1007/s00701-024-06229-1

**Published:** 2024-08-20

**Authors:** Shadi Al-Afif, Josef M. Lang, Arif Abdulbaki, Thomas Palmaers, Dirk Scheinichen, Omar Abu-Fares, Elvis J. Hermann, Joachim K. Krauss

**Affiliations:** 1 Department of Neurosurgery, Hannover Medical School, Hannover, Germany; 2 Department of Anaesthesiology and Intensive Care, Hannover Medical School, Hannover, Germany; 3 Institute of Diagnostic and Interventional Neuroradiology, Hannover Medical School, Hannover, Germany

**Keywords:** Clipping, PICA aneurysm, Semi-sitting position, Venous air embolism, Abbrevations, SAHsubarachnoid hemorrhage, CSFcerebrospinal fluid, VAEvenous air embolism, CTcomputer tomography, EtCO2end-tidal carbon dioxide, SSEPs somatosensory evoked potentials, ICUintensive care unit, PICAposterior inferior cerebellar artery, ASAAmerican Society of Anesthesiologists, PFOpatent foramen ovale, TTEtransthoracic echocardiography

## Abstract

**Background:**

The semi-sitting position offers advantages for surgeries in the posterior cranial fossa. However, data on its safety and effectiveness for clipping aneurysms in the posterior cerebral circulation are limited. This retrospective cohort study evaluates the safety and effectiveness of using the semi-sitting position for these surgeries.

**Methods:**

We conducted a retrospective study of 17 patients with posterior cerebral circulation aneurysms who underwent surgical clipping in the semi-sitting position in the Department of Neurosurgery at Hannover Medical School over a 10-year period.

**Results:**

The mean age at surgery was 62 years (range, 31 to 75). Fourteen patients were admitted with subarachnoid hemorrhage and 3 patients had incidental aneurysmas. Fifteen patients had PICA aneurysms, and two had aneurysms of the vertebral artery and the superior cerebellar artery, respectively. The median diameter of the aneurysms was 5 mm (range 3–17 mm). Intraoperative venous air embolism (VAE) occurred in 4 patients, without affecting the surgical or clinical course. VAE was associated with a mild decrease of EtCO2 levels in 3 patients and in 2 patients a decrease of blood pressure occurred which was managed effectively. Surgical procedures proceeded as planned in all instances. There were no complications secondary to VAE. Two patients died secondary to respiratory problems (not related to VAE), and one patient was lost to follow-up. Eleven of fourteen patients were partially or completely independent (Barthel index between 60 and 100) at a median follow-up duration of 13.5 months (range, 3–103 months).

**Conclusion:**

The semi-sitting position is a safe and effective technique for the surgical clipping of aneurysms in the posterior cerebral circulation. The incidence of VAE is comparable to that seen in tumor surgery. However, it is crucial for the surgical and anesthesiological team to be familiar with potential complications and to react immediately in case of an occurrence of VAE.

## Introduction

Aneurysms of the posterior cerebral circulation, apart from those located on the basilar artery tip, are rare and account for only 0.5–3% of all intracranial aneurysms [20, 22, 31]. The primary clinical manifestation in patients with these aneurysms usually is subarachnoid hemorrhage (SAH) [22]. Given their rarity and their close proximity to the brainstem and the cranial nerves their management is particularly challenging [25].

SAH resulting from the rupture of a cerebral aneurysm is a critical neurosurgical emergency that requires immediate treatment. One of the key strategies for treating SAH is the prompt and safe closure of the ruptured aneurysm to prevent potentially fatal re-rupture [14]. When microsurgical treatment is chosen, the surgeon must reflect on various issues when planning the surgical procedure. Patient positioning and an effective surgical approach are among the most crucial factors to be considered [17].

Several positions have been described for surgical clipping of aneurysms in the posterior cerebral circulation, including the supine position, prone position, and the three-quarter prone position (also known as the park bench position) [2, 18, 23, 29]. The semi-sitting position has been used with increasing frequency to manage various pathologies in the posterior cranial fossa, mainly for the resection of tumors, such as vestibular schwannomas, tumors in the pineal region, or tumors in the fourth ventricle [1, 3, 10, 13, 16, 26, 27], but it has not been applied commonly for vascular lesions of the posterior fossa.

The semi-sitting position offers several advantages, including improved drainage of blood and cerebrospinal fluid (CSF), which reduces the need for excessive suction, and it allows the surgeon to utilize both hands during dissection around critical neurovascular structures [8, 12]. It offers also other benefits such as reduced brain swelling and improved maneuverability around critical structures. However, several issues are associated with this position, including the risk of venous air embolism (VAE) and cardiac arrhythmias. Additionally, the technical dissection of critical neurovascular structures can be challenging when surgeons have to keep their arms elevated during the surgery.

There is only limited data available regarding both the applicability and the safety of the semi-sitting position for the clipping of aneurysms in the posterior circulation. Here, we report on our experience in a series of 17 patients.

## Methods

All patients who underwent clipping of cerebral aneurysms in the semi-sitting position over a 10-year period (between 2012 and 2022) in the Department of Neurosurgery at Hannover Medical School were included in this study. The protocol for this retrospective analysis involved reviewing all available clinical and imaging data, including documentation of the rehabilitation process.

A total of 17 patients who underwent clipping of cerebral aneurysms in the semi-sitting position were identified during the study period.

The study was conducted in compliance with the regulations of the local ethical committee at our institution. Informed consent was obtained from all individual participants included in the study. The patients consented to the publication of their protected health information.

### Preoperative management and surgical treatment

After admission, all patients underwent a comprehensive preoperative assessment, which included CT imaging and CT angiography. Additionally, patients presenting with acute hydrocephalus underwent CSF diversion either through ventricular or lumbar drainage. Preoperative digital subtraction angiographies were obtained for better demonstration of the aneurysm shape and morphology, and the vascular supply and dynamics. Subsequently, an interdisciplinary team consisting of a neurosurgeon and a neuroradiologist made a collaborative decision to determine the most appropriate form of treatment.

Surgery in the semi-sitting position was performed as outlined in previous studies [1, 11, 15, 24]. In summary, the preparation of all patients included the placement of a triluminal central line in the right atrium, a peripheral catheter in the radial artery, a peripheral venous catheter, and a bladder catheter. Intraoperative monitoring involved several measures, including transesophageal Doppler or echocardiography to detect VAE, pulse oximetry, invasive arterial blood pressure recording, and continuous measurement of end-tidal carbon dioxide (EtCO2) levels.

**Fig. 1:**
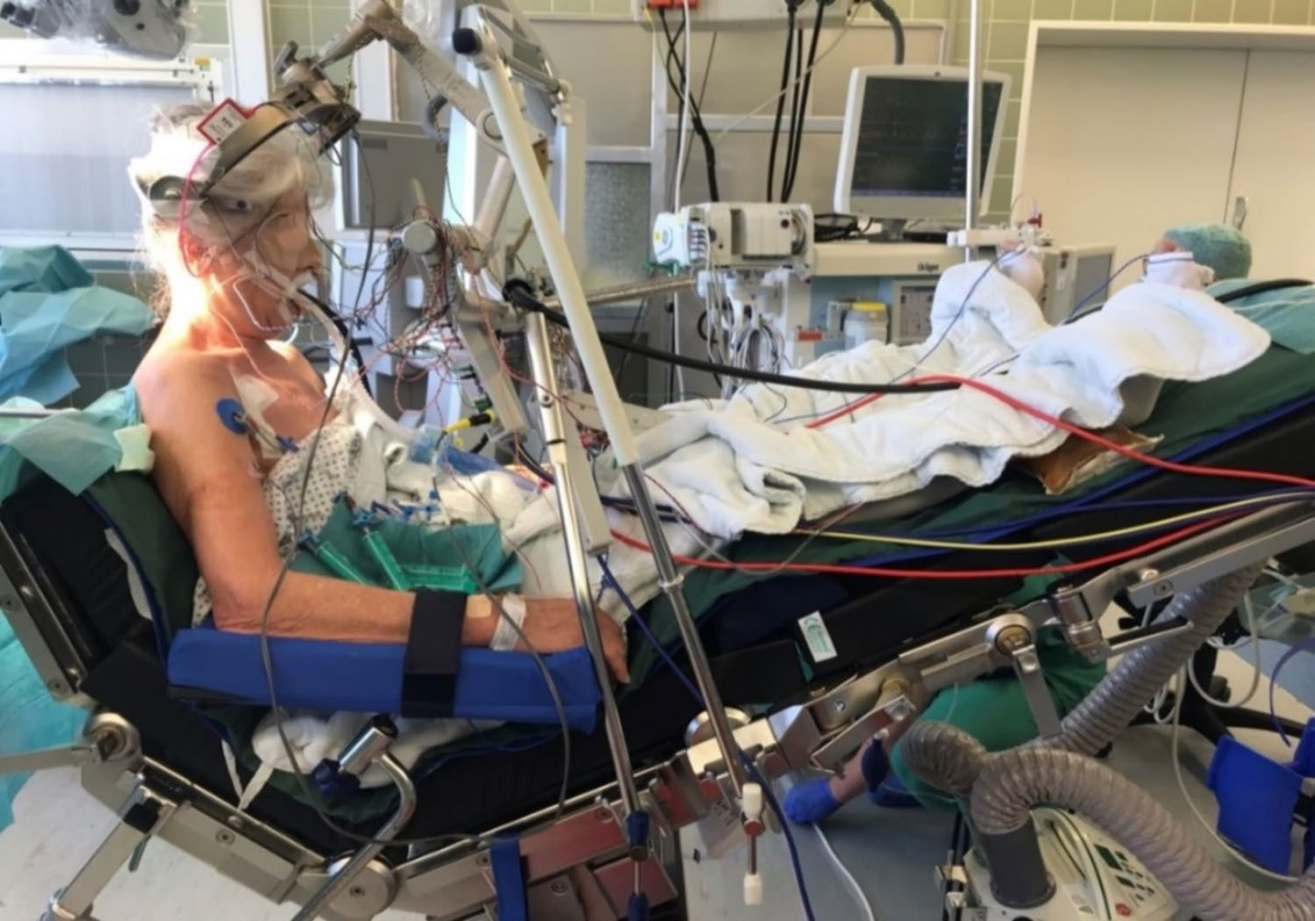
Demonstration of the semi-sitting position with the legs elevated above the level of the heart to minimize the risk of VAE

During the procedure, the patient was positioned supine on the operating table, with the head secured in a Mayfield clamp. The upper body and legs were gradually elevated by adjusting the operating table to achieve the semi-sitting position [1]. The patient's legs were positioned at or above heart level. The head was carefully flexed anteriorly while monitoring SSEPs, ensuring that a 2-finger space was maintained between the patient's chin and the sternal notch to prevent venous obstruction and facilitate jugular vein compression if VAE should occur. This would allow the surgeon to localize the air entry site. Special padded armrests were used to support the patient's arms during the procedure **(**Fig. 1**)**.

The primary surgical approach was a median craniotomy, which could be extended laterally depending on the exact location of the aneurysm. Saccular aneurysms were treated primarily by clip occlusion using one or more clips.

### Postoperative measurements

Following the surgery, all patients were transferred to the intensive care unit (ICU) for postoperative care and further treatment. Within 6 h after surgery, a postoperative cranial CT scan was obtained routinely to rule out any early surgical complications, such as hematoma or cerebral infarction. In case of relevant amounts of intracranial air depots, normobaric hyperoxia was installed as described in detail elsewhere [15]. Additionally, another cerebral digital subtraction angiography was performed within 3–10 days after surgery to assess the complete occlusion of the aneurysm.

In the early postoperative period, patients were closely monitored for the occurrence of cerebral vasospasms by cerebral Doppler sonography and clinical assessment.

### Follow-Up

Follow-up data were collected through semi-standardized interviews conducted with patients, their relatives, or their family doctors.

## Results

Out of the 17 patients 9 were women and 8 were men. The mean age at the time of surgery was 62 years (range, 31—75 years). The majority of patients (14/17) presented with SAH.

The severity of SAH at presentation, according to the Hess and Hunt classification, was as follows: four patients were classified as grade 2, four as grade 3, two as grade 4, and four as grade 5. Two patients had a history of previous SAH due to the rupture of an aneurysm at a different location. The medical history and the clinical state at admission are shown in Table 1.

**Table 1:** Demographic and clinical data of 17 patients with aneurysms in the posterior fossa operated in the semi-sitting position

	Age	Sex	BMI, kg/m^2^	Medical history	Clinical presentation
1	54	F	24	HTN, AF, COPD	SAH Hunt and Hess grade 5
2	60	M	30.4	HTN, CHD, hypercholesterolemia, DM, SM	SAH Hunt and Hess grade 3
3	51	M	26.2	CHD, COPD, SM	SAH Hunt and Hess grade 2
4	71	F	29.1	HTN, HF, asthma	SAH Hunt and Hess grade 5
5	74	F	23.9	HTN, COPD, SM	SAH Hunt and Hess grade 3
6	70	M	24.7	HTN, CHD, MR, CEA	SAH Hunt and Hess grade 2
7	75	M	24.8	HTN	SAH Hunt and Hess grade 2
8	65	F	24.9	HTN, stroke, COPD, SM	SAH Hunt and Hess grade 3
9	67	M	27.8	HTN, DM	SAH Hunt and Hess grade 5
10	39	M	27	Ulcerative colitis, cocaine addiction	SAH Hunt and Hess grade 2
11	71	M	19	HTN, COPD, DM, CHD, PAD, SM	SAH Hunt and Hess grade 4
12	64	M	24.8	HTN, DM, SM	SAH Hunt and Hess grade 5
13	63	F	25.5	None	SAH Hunt and Hess grade 3
14	55	F	24.9	COPD, history of SAH, rheumatoid arthritis, dissection of right VA	SAH Hunt and Hess grade 4
15	61	F	31.6	HTN, SM, obesity, Raynaud's syndrome, hypercholesterolemia	Incidental, headache
16	31	F	19.70	History of SAH with coiling of PICA-aneurysm Hunt and Hess grade 3. Reperfusion of the aneurysm 8 months after coiling, HTN, SM	Incidental, headache
17	53	F	24.2	MS, SM, depression	Incidental, headache

F = female, M = male, HTN = arterial hypertension, AF = atrial fibrillation, COPD = chronic obstructive pulmonary disease, DM = diabetes mellitus, H&H = Hunt and Hess grading, SM = smoking, HF = heart failure, MR = mitral regurgitation, CHD = coronary heart disease, CEA = carotid endarterectomy, MS = Multiple sclerosis:

Of the 14 patients presenting with SAH, 12 had ruptured aneurysms of the posterior inferior cerebellar artery (PICA). The other two patients had a ruptured aneurysm of the superior cerebellar artery, and a dissceting aneurysm of the vertebral artery, respectively. Of the 14 patients with SAH, intraventricular hematoma was observed in seven patients, intracerebellar hematoma in three, and subdural hematoma in two. All three patients with unruptured aneurysms had PICA aneurysms. Individual patient data are shown in Table 2.

**Table 2:** Surgical and intraoperative data

	Location of aneurysm	Size of aneurysm in mm	Intracranial hematoma on CT	EVD or LD	ASA score	Surgery duration, minutes	Amount of iv crystalloids, ml	Amount of iv colloids, ml
1	PICA	4 × 2	SAH,IVH	EVD	III	235	4500	500
2	PICA	3 × 4	SAH, IVH, ICB	EVD	IV	343	3560	0
3	PICA	4 × 4	SAH, SDH	-	II	188	4172	1500
4	SCA	2 × 5	SAH, ICB	EVD	IV	263	3546	0
5	PICA	7 × 6	-	EVD	III	203	4068	0
6	PICA	5 × 4	SAH, IVH, ICB,SDH	EVD	IV	172	1433	0
7	PICA	6 × 5	SAH, IVH	LD	III	92	2715	500
8	PICA	17 × 14	SAH	EVD	III	385	3873	1000
9	PICA	8 × 5	SAH	EVD	IV	431	4607	0
10	VA (dissecting aneurysm)	5 × 4	SAH	-	II	301	3565	500
11	PICA	3 × 4	SAH, IVH	EVD	IV	141	1000	1000
12	PICA	4 × 3	SAH, IVH	EVD	IV	265	1500	1000
13	PICA	7 × 5	SAH	EVD	III	120	3861	0
14	VA-PICA (dissecting aneurysm)	4 × 3	SAH, IVH	VP shunt	IV	119	1475	0
15	PICA	7 × 4	-	-	III	137	2881	500
16	PICA	5 × 3	-	-	II	149	2792	500
17	2 PICA aneurysms	3 × 3 & 7 × 5	-	-	II	231	4824	1500

SAH subarachnoid hemorrhage, H&H Hunt and Hess grading, PICA posterior inferior cerebellar artery, SCA superior cerebellar artery, IVH intraventricular hemorrhage, SDH subdural hematoma, ICB intracerebral bleeding, EVD external ventricular drainage, LD lumbar drainage, VA vertebral artery, VAE venous air embolism

Preoperative digital subtraction angiography identified a single aneurysm in 16/17 patients, while in one patient, two PICA aneurysms on the same side were detected and both of them were clipped (patient 17). The size of the aneurysms is also shown in Table 2 (median size of the largest diameter was 5 mm, ranging from 3 to 17 mm). In two patients, dissections of the vertebral artery were evident.

Six patients with SAH required intubation and mechanical ventilation before performing the angiography. Of the patients with SAH, eleven developed an acute hydrocephalus (placement of ventricular drainages was necessary in 10 patients, and lumbar drainage in one patient). A ventriculoperitoneal shunt had been placed in one instance with a previous SAH**.**

The scores of the American Society of Anesthesiologists (ASA) Physical Status Classification System were distributed as follows: IV in 7 patients, III in 6 patients, and II in 4 patients **(see also **Table 2**).**

### Intraoperative course

The median duration of surgery was 203 min, ranging from 92 to 431 min. On average, patients received 3.198 ml of crystalloid fluids during the procedure. Colloid fluids were administered in 10 patients, while blood transfusion was required in only one instance.

During surgery, there was a rupture of the aneurysm in two patients (both of them had presented with SAH), which was successfully controlled in both instances.

Adenosine was administered intraoperatively in 4 patients to induce arterial hypotension (without relevant complications).

### Incidence and severity of VAE

VAE was detected in 4 patients (23.5%) during intraoperative monitoring **(**Table 3**)**. The severity of VAE (according to the Tübingen grading scale [6]) was classified as grade 2 in 2 patients, and grade 3 and 4 in one patient, respectively. VAE was associated with a mild decrease of EtCO2 levels in 3 patients, by 2, 3 and 5 mmHg, respectively. In two patients, a significant decrease in systolic blood pressure of 20 and 40 mmHg was observed, respectively, which was successfully corrected using catecholamines and fluids. The source of the VAE was identified by the surgeon within the surgical site in all patients, and appropriate measures were implemented to ensure its proper control. The surgical procedure proceeded as planned in all instances.

**Table 3:** Occurrence of venous air embolism in 4 patients during surgery

Patient no	Grade of VAE	Decrease in EtCO2	Blood pressure decrease	Air aspiration from the iv central line	Complications related to VAE
9	2	3 mmHg	none	no	none
10	2	2 mmHg	none	yes	none
14	4	none	40 mmHg	no	none
17	3	5	20 mmHg	yes	none

EtCO2 End-tidal CO2

iv, intravenous

Postoperative CT scans did not reveal any complications related to VAE in the four patients.

### Postoperative course und follow-up

In all patients the surgery was completed successfully. There were no intraoperative complications apart from VAE. Subdural air depots were present in 12/17 patients, which were less than 1 cm (maximum anterior–posterior diameter) in 7 instances, and more than 1 cm in 5. Intraventricular air depots were present in 6/17 patients, which were mild (circumscribed depot one side only) in 2 instances, and more extensive in 4. These depots did not cause additional clinical symptoms. Three patients subsequently had asymptomatic subdural effusions of less than 1 cm (maximum anterior–posterior diameter), which all resolved spontaneously in the following. The median time of hospital stay was 27 days, ranging from 9 to 90 days, which coincided with the median time of stay in the ICU (26 days, ranging from 1 to 90 days; see Table 4. Six patients developed cerebral vasospasms within the first three weeks post-surgery and were treated with a protocol raising systolic arterial pressure and repeated intra-arterial vasospasmolysis using nimodipine.

**Table 4:** Postoperative course and follow-up

	Duration of hospital stay (days)	Duration of ICU stay (days)	Occurrence of vasospasm	Cerebral infarction	Other complications	Tracheostomy at discharge	VP shunt	Last follow up (months)	Barthel-Index
1	70	66	VA	no	Pneumonia	no	no	103	100
2	22	21	no	no	Pneumonia	yes	no	-	-
3	50	24	VA	no	Pneumonia	no	no	87	35
4	39	39	no	no	Pneumonia, VTE	yes	no	6	80
5	20	20	no	no	no	no	yes	17	65
6	23	23	VA and ACI right	Media infarction and cerebellar	Pneumonia, hemiparesis, aphasia	no	no	Death	-
7	25	21	no	no	no	no	yes	3	100
8	46	46	no	no	Pneumonia	yes	yes	38	85
9	51	50	VA	Cerebellar	Spastic tetraparesis	yes	yes	8	10
10	31	31	VA	Cerebellar	Rectal bleeding	no	no	10	90
11	90	90	no	no	Pneumonia, pleural effusion	yes	yes	Death	-
12	30	30	no	Cerebellar	Brain edema, pneumonia, pleural effusion, spastic tetraparesis	yes	yes	46	20
13	27	27	VA and ACI	Cerebellar and temporal	Pneumonia	yes	no	58	90
14	26	26		Cerebellar	Pneumonia	yes	already have	10	80
15	10	1	no	no	no	no	no	4	100
16	9	1	no	no	no	no	no	46	100
17	21	19	no	Cerebellar	no	no	no	4	60

VA = vertebral artery, ACI = Internal carotid artery

Three patients had cerebral infarctions related to vasospasm (patients 6, 9 and 13), whereas two other patients developed cerebral infarctions associated with vertebral artery dissection in addition to vasospasm (patients 10 and 14), while two patients had surgery-related cerebral infarction (patients 12 and 17). Postoperative angiography confirmed complete occlusion of the aneurysms, but in two patients compromised parent PICA flow was noted (patients 12 and 17).

Ten patients developed pneumonia during ICU stay but were successfully treated with antibiotics. Additionally, one patient developed lower extremity thrombosis. Out of 14 patients presenting with SAH, 8 underwent tracheostomy during the postoperative period in the ICU. One patient died during ICU stay due to severe respiratory failure, and another one passed away during the early phase of the rehabilitation due to severe pneumonia.

Six patients required ventriculoperitoneal shunts. All patients were referred to rehabilitation for further treatment.

Follow-up information was available in 14 of the 15 surviving patients. The median follow-up duration was 13.5 months (range 3–103 months). The Barthel index [19] of the individual patients at the time of the last follow-up is shown in Table 4**.** Eleven patients had a Barthel index between 60 and 100, indicating partially or completely independent functional outcome.

## Discussion

Our study shows that the semi-sitting position is safe and effective for the clipping of aneurysms in the posterior cerebral circulation. VAE occurred in about a fourth of patients without having a major impact on the intraoperative or postoperative course. A previous study has demonstrated previously that, although there is an increased burden on the surgeon upon the occurrence of VAE, the surgeon’s performance is not compromised when experienced neurosurgeons and anaesthesiologists collaborate [1].

There is very little experience using the semi-sitting positions for clipping of PICA and vertebral artery aneurysms [4, 5, 9, 17, 23, 28]. Thus far, this topic, in contrast to the renaissance of applying the semi-sitting position for tumor resection in the posterior fossa [1] has not been in the focus of attention and published literature is limited mainly to case reports and technical considerations.

We show here that the frequency of VAE in the semi-sitting position for the surgical clipping of incidental and ruptured cerebral aneurysm is similar to that observed in tumor surgery [1]. Remarkably, also induced arterial hypotension using adenosine can be performed, and the use of rapid ventricular pacing in this position has been described as well [9]. Remarkably, 3/4 of the patients with Hunt and Hess grade 5 in our study had a favorable outcome. While this might be co-incidental, it may also be related to the aggressive and early surgical treatment of the aneurysms.

The semi-sitting position may be used in both unruptured and ruptured aneurysms presenting with SAH. In two of the previously published case reports, the initial clinical presentation was SAH [9, 28], while in the third case report, hydrocephalus caused by a giant P3-aneurysm was the primary clinical presentation [5].

The occurrence of VAE and its impact on surgical outcome in patients with aneurysms of the posterior circulation has not been described in detail in the other studies concentrating on technical aspects of the semi-sitting position. In a study on 80 patients with vertebral artery and PICA aneurysms Pilipenko et al. used the semi-sitting position in 52 instances [23]. They reported a very low incidence of VAE (3.8%) but the methodology for monitoring VAE was not discussed in their study.

In a previous study, it was demonstrated that VAE detection rates are significantly dependent on the monitoring technique: VAE was detected in 40.5% of patients when transesophageal echocardiography (TEE) was used, as opposed to 11.7% when transthoracic Doppler echocardiography (TTDE) was utilized [1].

Krayenbühl and colleagues reporting on technical strategies to approach aneurysms of the vertebral and posterior inferior cerebellar arteries, mentioned that they occasionally use the semi-sitting position [17]. They indicated that they selected the prone position for patients with an average weight and a relatively long neck, particularly when a paramedian incision was planned. They opted for the semi-sitting position in patients who were obese with a short or thick neck. However, they did not provide details regarding the use of the semi-sitting position during aneurysm clipping especially regarding the incidence of VAEs and their impact on surgery and outcome.

Clipping of the aneurysms in our series using the semi-sitting position was successful in all instances which was also noted in the case reports published previously [4, 5, 9, 28]. A premature rupture in two patients was managed effectively without anaesthesiological compromise. In any case, however, surgeons should formulate a precise surgical strategy before employing this position. For instance, the semi-sitting position may not be suitable when bypass surgery is planned.

A crucial aspect is the importance of effective communication between surgeon and anaesthesiologist before and during the procedure. Unexpected events during surgery, such as severe VAE with deregulation of arterial blood pressure, can only be managed successfully with established protocols [1]. Otherwise, overcorrection of arterial hypotension could lead to premature rupture of the aneurysm or swelling of the cerebellum. The team should also be prepared for extreme situations such as the need for resuscitation, which can be performed after adjusting the operating table [8].

Intraoperative cardiac arrhythmias, which are more frequent in patients with SAH [7] were not encountered in our study in the semi-sitting position. This is important insofar, as the semi-sitting position itself has been recognized as a risk factor for cardiac arrhythmias, attributed to sudden arterial pressure drops leading to atrial tachyarrhythmia [6]. Certainly, severe and uncontrolled VAE is the primary cause of severe cardiac rhythms anomalies in this position, underscoring the importance of its effective prevention and management.

Cardiac right-to-left shunts, such as a patent foramen ovale (PFO), can lead to cerebral injury from paradoxical air embolism, even in small amounts [21, 30]. At our institution, we routinely screen patients preoperatively prior to planning surgery in the semi-sitting position for PFO using transthoracic echocardiography (TTE) with intravenous echo-contrast. If PFO or other shunts are detected, we generally opt for prone positioning. In emergency situations such as it is the case with SAH, TTE usually is not possible for logistic reasons. In such circumstances, we investigate the patient by using TEE directly after intubation. If a PFO is detected then we usually proceed with prone positioning as outlined above.

Applying the semi-sitting position in the presence of a PFO is controversial. While most surgeons would not take advantage of this position in such a case, others deem it acceptable [6, 16, 27].

While there was no intraoperative mortality, two patients succumbed during the postoperative period. Four other patients experienced unfavorable outcomes, with three of them not recovering functionally well. Remarkably, 3/4 of the patients with Hunt and Hess grade 5 in our study had a favorable outcome. While this might be co-incidental, it may also be related to the aggressive and early surgical treatment of the aneurysms. These poor long-term outcomes for ruptured PICA aneurysms echo findings from previous studies, where 63% of patients demonstrated poor outcomes at 1-year follow-up [31]. This is largely attributable to the proximity of these aneurysms to the brainstem and caudal nerves.

The main limitation of this study is the retrospective design with potential biases, such as selection bias. Second, the sample size with only 17 patients is relatively small.

## Conclusion

We conclude that the semi-sitting position can be used for clipping aneurysms of the posterior cerebral circulation when performed by experienced interdisciplinary teams.

## Data Availability

The authors confirm that data are available on reasonable requests.
